# Effects of the Modified Antitubercular Treatment Regimen on Renal Function in Patients With Chronic Kidney Disease and Pulmonary Tuberculosis: An Observational Study

**DOI:** 10.7759/cureus.37013

**Published:** 2023-04-01

**Authors:** Aakankshya Tripathy, Jyoti Prakash Sahoo, Manoranjan Pattnaik, Trupti R Swain

**Affiliations:** 1 Pharmacology, Srirama Chandra Bhanja (SCB) Medical College and Hospital, Cuttack, IND; 2 Pharmacology, Kalinga Institute of Medical Sciences, Bhubaneswar, IND; 3 Pulmonary Medicine, Srirama Chandra Bhanja (SCB) Medical College and Hospital, Cuttack, IND

**Keywords:** body mass index: bmi, egfr creatinine, kidney disease, tuberculosis, antitubercular drugs

## Abstract

Introduction: Chronic kidney disease (CKD) increases an individual's vulnerability to infections like tuberculosis. Doses of pyrazinamide and ethambutol are modified to treat such individuals. Additionally, the renal function tends to decline with advancing age. Therefore, it is crucial to study the effect of antitubercular drugs on renal function in young and elderly patients. The primary objective of this study was to determine the change in serum creatinine levels at six months from baseline in two study groups that included patients aged ≤50 and >50 years. The secondary objective was to determine changes in estimated glomerular filtration rate (eGFR) and BMI six months from baseline.

Methods: We recruited 40 patients with CKD and pulmonary tuberculosis from Srirama Chandra Bhanja (SCB) Medical College and Hospital, India. Each participant received the modified doses of antitubercular drugs. Their serum creatinine, eGFR, and BMI were assessed at baseline, two and six months. Participants had a mean age of 50.93±9.83 years.

Results: The median changes in serum creatinine and eGFR values from baseline were -0.19 and -0.23 mg/dl and 4.16 and 3.93 ml/min/m^2^ for the two study groups, respectively. Furthermore, the differences in BMI from baseline were 1.91 and 2.14 kg/m^2^, respectively, for the two groups. The renal function was found to be improved after six months of treatment with modified antitubercular drugs. The intergroup comparisons were not statistically significant.

Conclusion: We conclude that the modified regimen helps effectively cure pulmonary tuberculosis and significantly improves renal function in CKD patients. Further studies are required to generalize these findings.

## Introduction

Chronic kidney disease (CKD) has mushroomed rapidly in eastern Indian states in the last decade [1]. Such immunocompromised conditions expedite other infectious diseases like tuberculosis (TB). Owing to the immunosuppressive effect of uremia, patients with CKD are at a high risk of developing tuberculosis [2]. In India, the incidence of pulmonary tuberculosis in patients with CKD is 10.5% [3]. As per the National Institute for Health and Care Excellence guidelines, the relative risk for developing active tuberculosis is 10%-25% in patients with advanced stages of CKD [4]. The renal parameters like serum creatinine and estimated glomerular filtration rate (eGFR) show an illustrious transition after 50 years of age. Antitubercular treatment (ATT) significantly sways the renal function as many antitubercular drugs are excreted through the renal route. Consequently, in patients with CKD, an improper dosage of ATT culminates in either therapeutic failure or burgeoned side effects [5].

As CKD is a pressing public health issue in low- and high-income countries, the TB-CKD association deserves closer scrutiny. Harnessing the appropriate treatment for TB patients with CKD is paramount for reducing morbidity and mortality [5-8]. Current guidelines for first-line antitubercular drugs advocate that dosages of ethambutol and pyrazinamide should be adjusted according to one’s renal function and body weight. Still and all, no change in the dosage is necessary for patients with mild renal insufficiency [6,9]. We mapped this study out to evaluate the effect of a modified ATT regimen on renal function in CKD patients with newly diagnosed pulmonary tuberculosis.

## Materials and methods

This prospective observational study was conducted from 15 February 2020 to 31 December 2021. We screened the patients with CKD visiting the pulmonary medicine outpatient department of Srirama Chandra Bhanja (SCB) Medical College and Hospital, Cuttack, for diagnosis of tuberculosis. Inclusion criteria were as follows: individuals of either gender >18 years of age, a known case of CKD with baseline eGFR <60 ml/min/m^2^ and a newly diagnosed pulmonary tuberculosis case confirmed by a positive sputum smear. The exclusion criteria of the study were as follows: having extra-pulmonary tuberculosis, having chronic liver disease, any cardiovascular, metabolic, or endocrine disorders, end-stage renal disease (ESRD) with eGFR ≤15 ml/min/m^2^, pregnant or lactating women, renal transplant recipients, and not giving written informed consent for participation in the study. We got an approval from the Institutional Ethics Committee (IEC), SCB Medical College (IEC application no. 136) before study initiation.

We enrolled the individuals fulfilling the study's predefined criteria and recorded the sociodemographic and clinical parameters at the baseline visit. According to the age at the baseline visit, the study population was assorted into two groups. Groups A and B included participants with ages ≤50 and >50 years, respectively. The primary objective was to assess the change in serum creatinine levels at six months from baseline in the two study groups. The secondary objective was determining changes in eGFR and BMI six months from the baseline in the two study groups. We calculated eGFR using the Modification of Diet in Renal Disease (MDRD) formula [10]. All the participants were followed up at two and six months from the baseline visit. They all underwent sputum smear examinations at baseline and follow-up visits. The doses of pyrazinamide and ethambutol were modified according to the eGFR of the participant. They were assessed for the clinical and microbiological cure of pulmonary TB vis-à-vis the renal outcomes, i.e., serum creatinine and eGFR.

We applied the Shapiro-Wilk test to check the normality of data distribution before any analysis. The categorical and continuous variables were expressed in frequency (%) and median (interquartile range). Wilcoxon’s signed-rank test was performed to analyze the primary and secondary objectives. For the statistical analysis of the data and generation of plots, we used the R software (version 4.1.1) [11].

## Results

We screened 72 patients with CKD and newly diagnosed pulmonary tuberculosis for this study. Twelve patients did not meet the study criteria, 2 did not provide consent, and the rest 58 were enrolled. Eighteen patients were lost to follow-up. Hence, 40 participants completed the study and were included in the final analysis. The baseline demographic and clinical parameters of the study population are shown in Table 1. The participants had a mean age of 50.93±9.83 years. The median serum creatinine and eGFR values at the baseline visit were 2.06 mg/dl and 34.92 ml/min/m^2^, respectively.

**Table 1:** Baseline sociodemographic and clinical parameters of the study population BMI: body mass index; eGFR: estimated glomerular filtration rate Categorical and continuous data are expressed as n (%) and median (interquartile range), respectively.

	Total (n=40)	Group A (n=20)	Group B (n=20)
Male	20 (50.00%)	10 (50.00%)	10 (50.00%)
Age, mean±SD	50.93±9.83	41.70±3.18	60.15±3.03
Age, median (range)	51.50 (41.00-60.25)	41.00 (39.75-44.00)	60.50 (57.75-63.00)
Marital status			
Married	36 (90.00%)	16 (80.00%)	20 (100.00%)
Single	4 (10.00%)	4 (20.00%)	0
Education			
Educated	32 (80.00%)	18 (90.00%)	14 (70.00%)
Uneducated	8 (20.00%)	2 (10.00%)	6 (30.00%)
Socioeconomic status			
Upper middle	2 (5.00%)	2 (10.00%)	0
Lower middle	4 (10.00%)	2 (10.00%)	2 (10.00%)
Upper lower	6 (15.00%)	2 (10.00%)	4 (20.00%)
Lower	28 (70.00%)	14 (70.00%)	14 (70.00%)
Weight (kg)	39.00 (33.00-45.25)	39.00 (35.50-46.25)	38.50 (32.50-43.25)
BMI (kg/m^2^)	17.53 (16.28-18.26)	17.36 (15.75-18.24)	17.72 (17.19-18.26)
Serum creatinine (mg/dl)	2.06 (1.92-2.37)	2.06 (1.87-2.21)	2.07 (1.95-2.46)
eGFR (ml/min/m^2^)	34.92 (29.43-36.65)	35.95 (33.24-39.90)	33.28 (27.20-35.28)

Clinical parameters of the study population at each time point of assessment are shown in Table 2. At the baseline visit, median serum creatinine values for Groups A and B were 2.06 and 2.07, respectively (p = 0.93). At six months, the serum creatinine values of both the study groups were 1.88. The median changes from baseline were -0.19 (-0.24 to -0.10) and -0.23 (-0.27 to -0.15), respectively. An abatement in serum creatinine was discerned in both study groups. The between-group comparison was not statistically significant (p = 0.20).

**Table 2:** Clinical parameters of the study population Data are expressed as median (interquartile range).

	Baseline	2 months	6 months	Change from baseline	p-value
Serum creatinine (mg/dl)					
Group A (n=20)	2.06 (1.87 to 2.21)	1.99 (1.85 to 2.12)	1.88 (1.80 to 2.03)	-0.19 (-0.24 to -0.10)	0.20
Group B (n=20)	2.07 (1.95 to 2.46)	2.02 (1.85 to 2.34)	1.88 (1.72 to 2.26)	-0.23 (-0.27 to -0.15)	
eGFR (ml/min/m^2^)					
Group A (n=20)	35.95 (33.24 to 39.90)	36.61 (34.64 to 40.16)	39.53 (36.54 to 42.51)	4.16 (2.25 to 5.25)	0.88
Group B (n=20)	33.28 (27.20 to 35.28)	33.86 (28.86 to 37.51)	36.80 (29.84 to 40.53)	3.93 (2.94 to 5.09)	
BMI (kg/m^2^)					
Group A (n=20)	17.36 (15.75 to 18.24)	17.89 (16.79 to 18.98)	19.26 (17.75 to 20.45)	1.91 (1.64 to 2.58)	0.61
Group B (n=20)	17.72 (17.19 to 18.26)	18.61 (18.12 to 19.31)	19.77 (19.21 to 20.71)	2.14 (1.78 to 2.59)	

**Figure 1:**
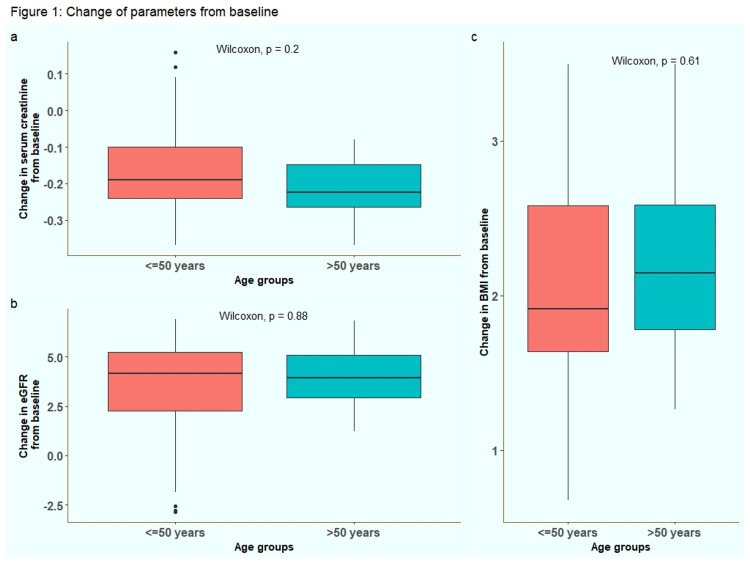
Change in (a) serum creatinine, (b) eGFR and (c) BMI from baseline BMI: body mass index; eGFR: estimated glomerular filtration rate

Baseline eGFR values were 35.95 and 33.28 for groups A and B, respectively (p = 0.34). Median changes from baseline were 4.16 (2.25 to 5.25) and 3.93 (2.94 to 5.09), respectively. Although there was an ascension in the eGFR of both the study groups, the inter-group difference was not statistically significant (p = 0.88). Similarly, the BMI in both groups had increased by 1.91 (1.64 to 2.58) and 2.14 (1.78 to 2.59), respectively. Nonetheless, the between-group comparison was not statistically significant (p = 0.61). The study objectives assessing the changes in study parameters are shown in Figure 1.

## Discussion

This observational study ascertained the effect of modified ATT on the renal parameters like serum creatinine, eGFR, and BMI of CKD patients aged ≤50 and >50 years with pulmonary tuberculosis. For the entire study duration, all participants received modified ATT according to their renal function and age. The doses of pyrazinamide and ethambutol were modified according to their eGFR. In this study, the diagnoses of pulmonary tuberculosis were based on a combination of clinical, radiographical, microbiological, and histopathological examinations.

A reduction in the serum creatinine level was found in both study groups, which was in concord with the study conducted by Li et al. [12]. Nevertheless, the between-group comparison was not statistically significant (p = 0.20). Strong adherence of the participants to the modified ATT regimen helped improve renal function along with the microbiological cure of tuberculosis infection. The uptick of eGFR values in the study population was not statistically significant (p = 0.88). This finding was consistent with that of the study by Li et al. [12]. The change in BMI from the baseline was not statistically significant (p = 0.61). This finding contrasted with that of Saito et al. [6]. Most of the study participants belonged to the lower socioeconomic class. The probable reasons for their poor nutritional status might be the lack of disease awareness, environmental hygiene, and adequate food and water supply.

However, the results of our study should be analyzed with a few limitations. First, a higher attrition rate, possibly because of the pandemic, reduced the study population. Second, excluding patients other than newly diagnosed pulmonary TB cases caused a further reduction in the study population. Third, we could not obtain extensive data regarding co-morbidities and other concomitant medications. The reno-protective drugs contributed to the improvement in renal function. We did not analyze the contribution of those drugs.

## Conclusions

Our study concludes that the modified ATT regimen helps effectively cure pulmonary tuberculosis and significantly improves the renal function in CKD patients with newly diagnosed pulmonary tuberculosis, regardless of the demographic parameters. However, a more extensive study population and the inclusion of CKD staging are warranted to generalize the findings of this study.
